# Inhibition of PTP1b in the amygdala reduces food intake, body weight and modulates glycemic homeostasis in obese rats

**DOI:** 10.1186/1758-5996-7-S1-A143

**Published:** 2015-11-11

**Authors:** Natália Ferreira Mendes, Gisele de Castro, Mário José Abdalla Saad, Patrícia de Oliveira Prada

**Affiliations:** 1 Universidade Estadual de Campinas, Sorocaba, Brazil

## Background

The control of food intake depends, in part, of the action and signaling of hormones, such as insulin in hypothalamic neurons. In obesity, inflammatory cytokines activate protein phosphatases, such as protein tyrosine phosphatase 1B (PTP1B). PTP1B interacts with the insulin receptor (IR) and the insulin receptor substrate (IRS1), inhibiting them in hypothalamus. Other brain regions, that are part of the dopaminergic reward system, such as the amygdala, participate of the control of energy balance in parallel to the hypothalamus. Insulin exerts its anorexigenic effect also through the amygdala and, in obesity, this effect is abolished. However, it is not known whether PTP1B is expressed and participates of the regulation of insulin signaling in amygdala.

## Aim

To investigate whether PTP1B expression is increased in the amygdala of diet induced obese (DIO) rats and whether the inhibition of PTP1B has any effect on energy metabolism or on insulin signaling, or action in the central nucleus of the amygdala (CeA) in DIO rats.

## Materials and methods

Male Wistar rats with 8 weeks old were divided into two groups: Chow (n=25) fed with standard rodent chow, and DIO (n=25), that received high fat diet, both for more 8 weeks. To assess PTP1B protein expression, 5 animals from each group were anaesthetized and, then, performed the dissection of the CeA. The other 20 animals were underwent to stereotactic surgery for implantation of the cannula in CeA. After recovery, the animals were treated with sense and antisense oligonucleotide (ASO) for 7 days, to inhibit the expression of PTP1B in CeA, giving rise to the subgroups: Chow+Sense, Chow+ASO, DIO+Sense e DIO+ASO. During treatment, the parameters evaluated were blood glucose and body weight. At the end of treatment was conducted the euthanasia of animals for extraction of CeA.

## Results

Animals fed high fat diet had greater body weight gain, insulin resistance and increased PTP1B expression and activation in CeA compared to Chow group. When treated with ASO, DIO animals showed reduced expression of PTP1B in CeA, loss of body weight and food intake, and decrease in blood glucose levels, reaching values similar to those presented by the animals of the Chow group.

## Conclusion

These data suggest that the inhibition of PTP1B in the amygdala improved glucose homeostasis and reduced food intake and body weight in obese rats.

